# Study on viscoelastic-plastic creep model of rock based on creep parameter degradation

**DOI:** 10.1371/journal.pone.0355851

**Published:** 2026-08-12

**Authors:** Erjian Wei, Yuexian Pang, Xiaobing Ma

**Affiliations:** 1 School of Architectural Engineering, Henan University of Industry Technology, Nanyang, China; 2 Ministry of Education Demonstration Construction Project Virtual Simulation Training Base, Nanyang, China; Henan Polytechnic University, CHINA

## Abstract

Establishing a reasonable rock creep model is of great significance for analyzing the creep characteristics of rock and predicting the long-term stability of engineering rock mass. Based on the analysis of the nonlinear creep characteristics of rock, this paper innovatively proposes an unsteady Kelvin body that reflects the nonlinear characteristics of the attenuation creep stage. Based on the negative exponential function relationship between the viscosity coefficient and the stress and time, a nonlinear viscoplastic body that reflects the nonlinear characteristics of the accelerated creep stage is proposed. A viscoelastic-plastic creep model of rock considering the deterioration of creep parameters is constructed, and the creep equations of the creep model under one-dimensional stress state and three-dimensional stress state are derived. The model in this paper is used to fit and analyze the creep test data of sandstone and carbonaceous shale under different confining pressures. The results show that the fitting degree of the test curve and the model curve is good, with the coefficient of determination R^2^ generally above 0.98 and low RMSE/MAE values, indicating that the rock creep model considering the deterioration of creep parameters established in this paper can well describe the creep mechanical characteristics of rock. At the same time, the fitting effects of the model, the classical Nishihara model and the fractional Nishihara model are compared to further illustrate the correctness and advancement of the established model. The research in this paper not only enriches the theory of rock creep model, but also has certain theoretical reference value for the study of long-term stability of rock mass engineering.

## 1. Introduction

Rock itself has the inherent characteristics of creep. Many engineering examples have confirmed that the long-term safety and stability of rock mass engineering are closely related to its creep properties [1–4]. In particular, some large-scale projects related to the national economy and people’s livelihood, such as water conservancy and hydropower projects, tunnel projects, deep underground caverns, and mine high slope projects, have a long service life [5–9]. If the creep effect is ignored, it may cause major accidents in the instability and failure of these projects. Therefore, it is very important to study the creep characteristics of rock, and the rock creep model is the core research content of creep characteristics [10–16]. Establishing a reasonable rock creep model is of great significance for analyzing the creep characteristics of rock and predicting the long-term stability of engineering rock mass.

At present, many researchers have done a lot of research on rock creep models [17–23], especially in the non-accelerating stage of rock creep under low stress state. The study of creep characteristics has formed a systematic and mature theory, and some classical creep mechanics models such as Maxwell model, Kelvin model, Burgers model and Nishihara model have been established [24–29]. These models can well describe the first two stages of rock creep. However, the components of these models are linear and cannot well describe the nonlinear creep process of rock [30–33]. At present, many researchers have nonlinearly improved the models to describe the nonlinear accelerated creep process of rock [34–37]. Zhu et al. [38] based on fractional calculus theory and creep compliance substitution method, a transversely isotropic nonlinear creep model considering damage and rock strata angle is established based on the element combination model. Wang et al. [39] established a creep damage model of soft rock based on the new evolution equation of viscosity coefficient at each stage of soft rock creep under the influence of synergistic damage effect and the improved Newtonian element. Based on the Norton power law type and damage factor, Moghadam et al. [40] proposed an elastic-viscoplastic constitutive model to describe the expansion, short-term failure and long-term failure of rock salt during creep, which is used to study the time-dependent performance of rock salt underground gas storage caverns. Based on Nishihara model, Weibull distribution function and Perzyna viscoplastic theory, Chen et al. [41] established an improved viscoelastoplastic creep model which can describe the whole process of rock creep failure. This model can better reflect the relationship between rock creep deformation and damage, and make up for the defect that Nishihara model can not describe accelerated creep. Based on the framework of continuum damage mechanics, Nedjar et al. [42] established a three-dimensional phenomenological model that can describe the long-term creep of gypsum rock materials by using the viscoelastic coupling method, and gave a set of numerical simulations to prove the feasibility of the proposed model.

The above research has played a positive role in improving the theory of rock creep model [20,43,44]. However, after analysis and thinking, it is found that there is still a problem that most of these nonlinear improvements to the model are only carried out in the accelerated creep stage. In fact, the rock does not show nonlinear characteristics only in the accelerated creep stage, and it also shows obvious nonlinear characteristics in the attenuation creep stage. Therefore, when establishing the rock creep model, it is necessary to consider both the nonlinear characteristics of the accelerated creep stage and the nonlinear characteristics of the attenuation creep stage, so as to describe the creep behavior of the rock more accurately. Therefore, the creep parameters of these two stages should not be constant, but should be aging deterioration. It is worth noting that although the existing research has involved the nonlinearity of decay creep, most of the improvements mainly focus on the acceleration stage, and the unified description of the parameter degradation behavior at different stages is not sufficient. The focus of this paper is to give a complete theoretical framework from the unified perspective of parameter degradation over time. Based on this, this paper will start from the analysis of the nonlinear characteristics of rock creep process, put forward the unsteady Kelvin body reflecting the characteristics of the attenuation creep stage and the nonlinear viscoplastic body reflecting the characteristics of the accelerated creep stage, and then establish the rock viscoelastoplastic creep model based on the deterioration of creep parameters, and derive its three-dimensional creep equation, and then use the creep test data to verify the applicability and rationality of the model. The research in this paper not only enriches the theory of rock creep model, but also provides theoretical reference value for engineering application.

## 2. Establishment of creep model

### 2.1. Characteristics of rock creep process

**Fig 1:**
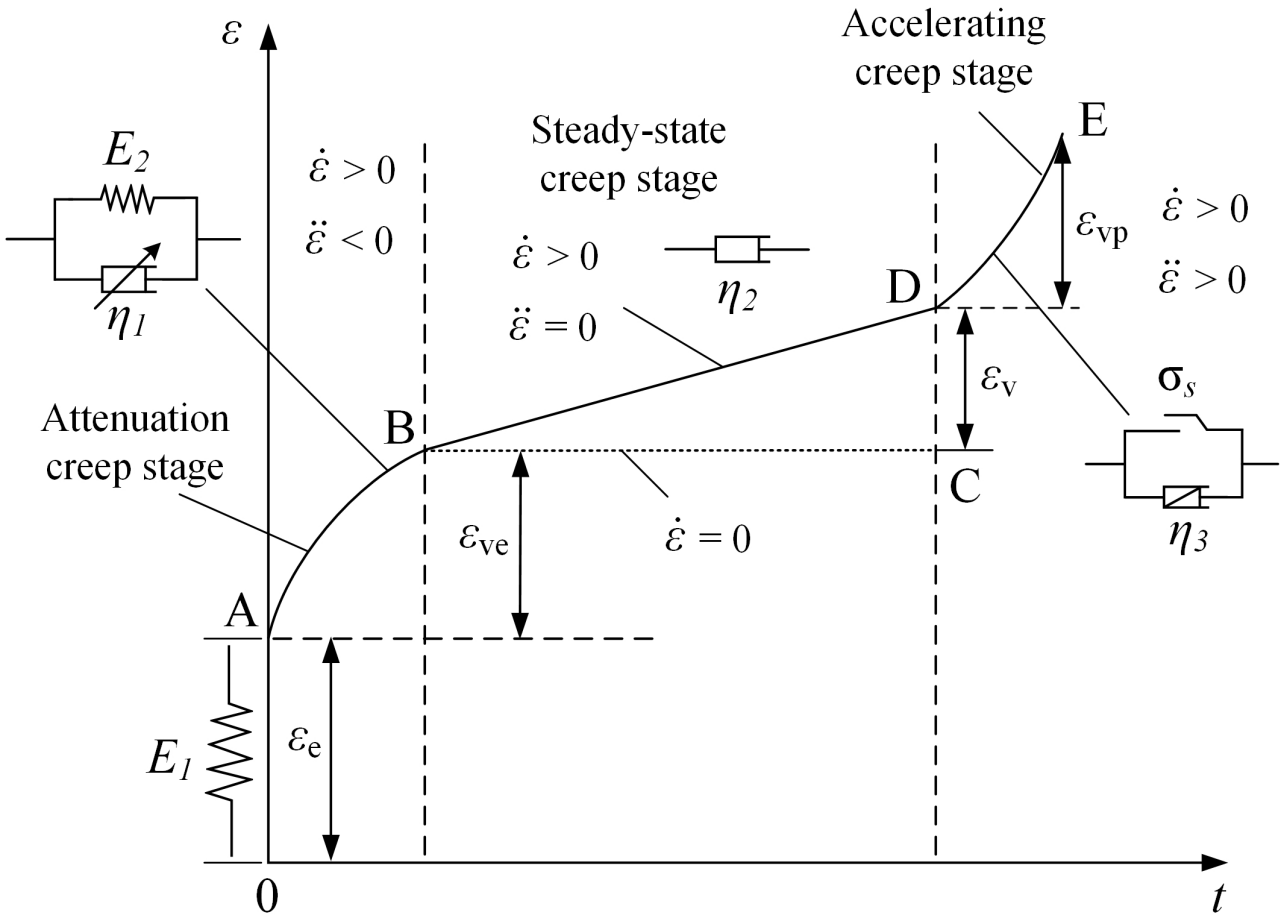
Characteristics of rock creep process.

The rock creep process is shown in Fig 1.

When the applied load is less than the long-term strength of the rock, the creep process of the rock is shown in the curve ABC in Fig 1. Instantaneous elastic deformation occurs at the moment of loading, and then enters the attenuation creep stage. The creep deformation increases continuously, while the creep rate decreases continuously, showing nonlinear attenuation characteristics. At a certain moment, the creep rate tends to zero, and the strain is stable at a certain value. The creep process of this case is divided into attenuation creep stage (such as curve AB) and stable creep stage (such as curve BC).

When the applied load exceeds the long-term strength of the rock, the creep process of the rock is shown in the curve ABDE in Fig 1. In the early stage of loading, the same deformation is produced, that is, instantaneous elastic deformation and nonlinear attenuation creep deformation. From a certain moment, the creep rate of the rock decays to a certain value and remains constant, and the creep deformation continues to increase. When it reaches a certain moment, the creep rate of the rock increases continuously, the nonlinear accelerated creep is significant, and the rock is quickly destroyed. The creep process in this situation encompasses a complete three-stage creep process: the attenuation creep stage (such as curve AB), the constant velocity creep stage (such as curve BD), and the accelerated creep stage (such as curve DE).

Here, a concept is explained. The above-mentioned stable creep stage and the constant creep stage are collectively referred to as the steady-state creep stage in many literatures, because both describe the steady-state creep process. The difference is that the stable creep focuses on indicating that the creep rate is very small, close to zero, and the creep of the rock is not obvious, while the constant creep focuses on emphasizing the significant creep of the rock.

### 2.2. Unsteady creep parameter Kelvin body

**Fig 2:**
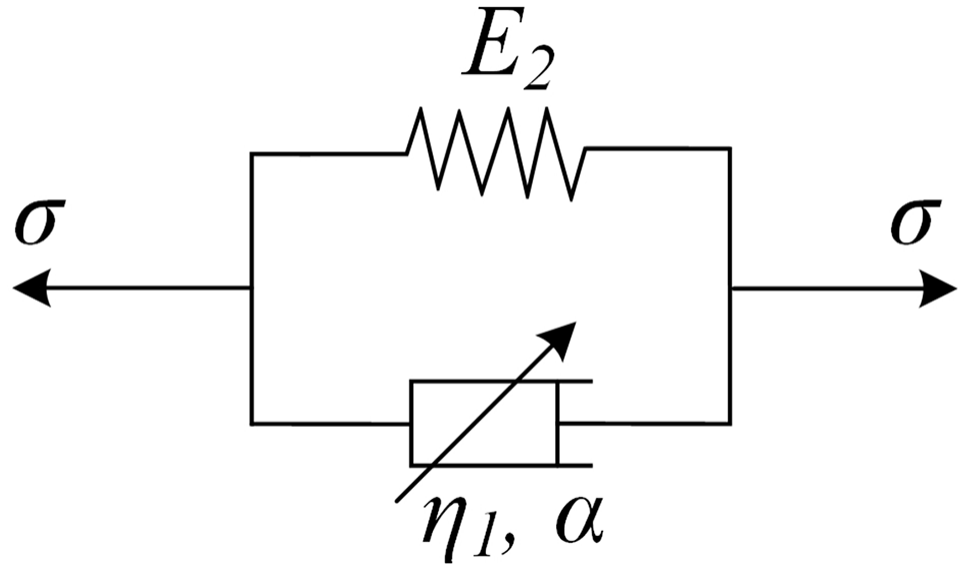
Unsteady creep parameter Kelvin body.

It can be seen from Fig 1 that the curve AB of the rock attenuation creep stage shows obvious nonlinear characteristics. It is difficult to accurately describe its creep behavior by using the traditional Kelvin body, mainly because the creep parameters in the traditional Kelvin body are constant and do not change with time. Therefore, the creep parameters of the rock attenuation creep stage should be unsteady, and should be continuously damaged and deteriorated with time. Based on this, this paper considers that the viscosity coefficient in the Kelvin model decreases with time. It is assumed that the viscosity coefficient and the creep time satisfy the power function relationship, so as to establish the Kelvin model of the unsteady creep parameters. The model diagram is shown in Fig 2.

It is assumed that the viscosity coefficient satisfies the following relationship:


$$\begin{array}{c} \eta =\eta_{\text{1}}{\left(1+\frac{t}{t_{\text{0}}}\right)}^{-\alpha } \end{array}$$ (1)

In the formula, *η* is the viscosity coefficient of the unsteady Kelvin body, *η*_*1*_ is the initial viscosity coefficient, *t* is time, *t*_0_ is the unit time, and *α* is a constant representing the degradation index of the viscosity coefficient. A higher α indicates a more rapid decrease in creep resistance during the decelerating stage.

The constitutive equation of the unsteady creep parameter Kelvin body is:


$$\begin{array}{c} \sigma =E_{\text{2}}\varepsilon_{ve}+\eta {\dot{\varepsilon }}_{ve}=E_{\text{2}}\varepsilon_{ve}+\eta_{\text{1}}(t/t_{\text{0}}+1{)}^{-\alpha }{\dot{\varepsilon }}_{ve} \end{array}$$ (2)

In the formula, *σ* is the stress, *E*_*2*_ is the elastic modulus of the unsteady Kelvin body, and *ε*_*ve*_ is the viscoelastic strain.

Solving the differential equation of Equation (2), we can get:


$$\begin{array}{c} \varepsilon_{ve}=\frac{\sigma }{E_{\text{2}}}+C\;exp\left(-\frac{E_{\text{2}}(t/t_{\text{0}}+1{)}^{\alpha +1}}{\eta_{\text{1}}(\alpha +1)}\right) \end{array}$$ (3)

When t=0, $\varepsilon_{ve}=0$, substituting this initial condition into Equation (3), the integration constant *C* can be obtained as:


$$\begin{array}{c} C=-\frac{\sigma }{E_{\text{2}}}exp\left(\frac{E_{\text{2}}}{\eta_{\text{1}}(\alpha +1)}\right) \end{array}$$ (4)

By substituting Equation (4) into Equation (3), we can obtain:


$$\begin{array}{c} \varepsilon_{ve}=\frac{\sigma }{E_{\text{2}}}+\left(-\frac{\sigma }{E_{\text{2}}}exp\left(\frac{E_{\text{2}}}{\eta_{\text{1}}(\alpha +1)}\right)\right)exp\left(-\frac{E_{\text{2}}(t/t_{\text{0}}+1{)}^{\alpha +1}}{\eta_{\text{1}}(\alpha +1)}\right) \end{array}$$ (5)

From Equation (5), we can get:


$$\begin{array}{c} \varepsilon_{ve}=\frac{\sigma }{E_{\text{2}}}-\frac{\sigma }{E_{\text{2}}}exp\left(\frac{E_{\text{2}}}{\eta_{\text{1}}(\alpha +1)}\left(1-{\left(1+t/t_{\text{0}}\right)}^{\alpha +1}\right)\right) \end{array}$$ (6)

The creep equation of the unsteady creep parameter Kelvin body can be obtained by simplifying Equation (6):


$$\begin{array}{c} \varepsilon_{ve}=\frac{\sigma }{E_{\text{2}}}\left(1-exp\left(\frac{E_{\text{2}}(1-(1+t/t_{\text{0}}{)}^{\alpha +1})}{\eta_{\text{1}}(\alpha +1)}\right)\right) \end{array}$$ (7)

### 2.3. Nonlinear viscoplastic body

**Fig 3:**
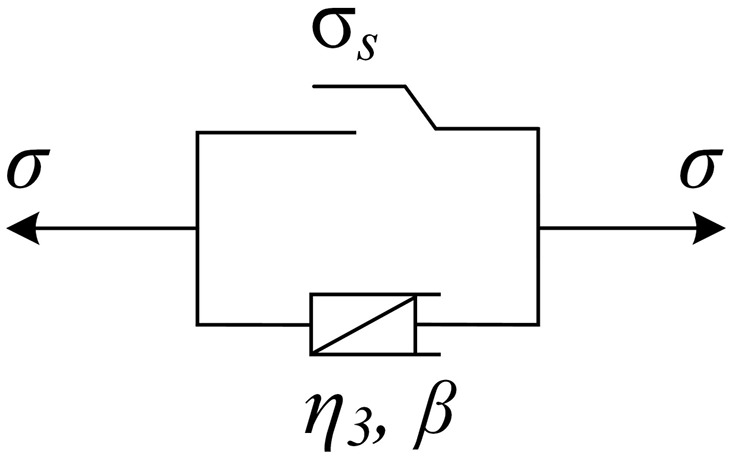
Nonlinear viscoplastic body.

When the applied load exceeds the long-term strength of the rock, after experiencing attenuation creep and steady-state creep, the rock will rapidly enter the accelerating creep stage. At this point, the strain and strain rate of the rock will increase sharply, showing obvious nonlinear characteristics. The microcracks in the rock will rapidly develop and form macroscopic cracks, and the damage of the rock will also increase sharply. At this time, the nonlinear viscoplastic body can be used to describe the nonlinear creep characteristics of the rock. The nonlinear viscoplastic body is shown in Fig 3.

In this process, the viscosity coefficient of rock decreases rapidly with stress and time. It is assumed that the viscosity coefficient satisfies the negative exponential function relationship with stress and time, which can be expressed as follows:


$$\begin{array}{c} \eta =\eta_{\text{3}}e^{-\beta (\sigma -\sigma_{s})t} \end{array}$$ (8)

In the formula, *η* is the viscosity coefficient of the nonlinear viscoplastic body, *η*_*3*_ is the initial viscosity coefficient, *σ* is the stress, *σ*_*s*_ is the long-term strength, and *β* is a constant. The constitutive equation of nonlinear viscoplastic body is:


$$\begin{array}{c} \sigma -\sigma_{s}=\eta {\dot{\varepsilon }}_{vp} \end{array}$$ (9)

Substituting Equation (8) into Equation (9), we can get:


$$\begin{array}{c} {\dot{\varepsilon }}_{vp}=\frac{\sigma -\sigma_{s}}{\eta_{\text{3}}}\cdot e^{\beta (\sigma -\sigma_{s})t} \end{array}$$ (10)

By integrating both sides of Equation (10), we can get:


$$\begin{array}{c} \varepsilon_{vp}=\frac{\text{1}}{\beta \eta_{\text{3}}}exp\left(\beta (\sigma -\sigma_{s})t\right)+C \end{array}$$ (11)

When t=0, $\varepsilon_{vp}=0$, substituting this initial condition into Equation (11), the integration constant *C* can be obtained as:


$$\begin{array}{c} C=-\frac{\text{1}}{\beta \eta_{\text{3}}} \end{array}$$ (12)

Therefore, the creep equation of nonlinear viscoplastic body is:


$$\begin{array}{c} \varepsilon_{vp}=\frac{\text{1}}{\beta \eta_{\text{3}}}\cdot \left(exp\left(\beta (\sigma -\sigma_{s})t\right)-1\right) \end{array}$$ (13)

### 2.4. Establishment of viscoelastic-plastic creep model of rock

**Fig 4:**
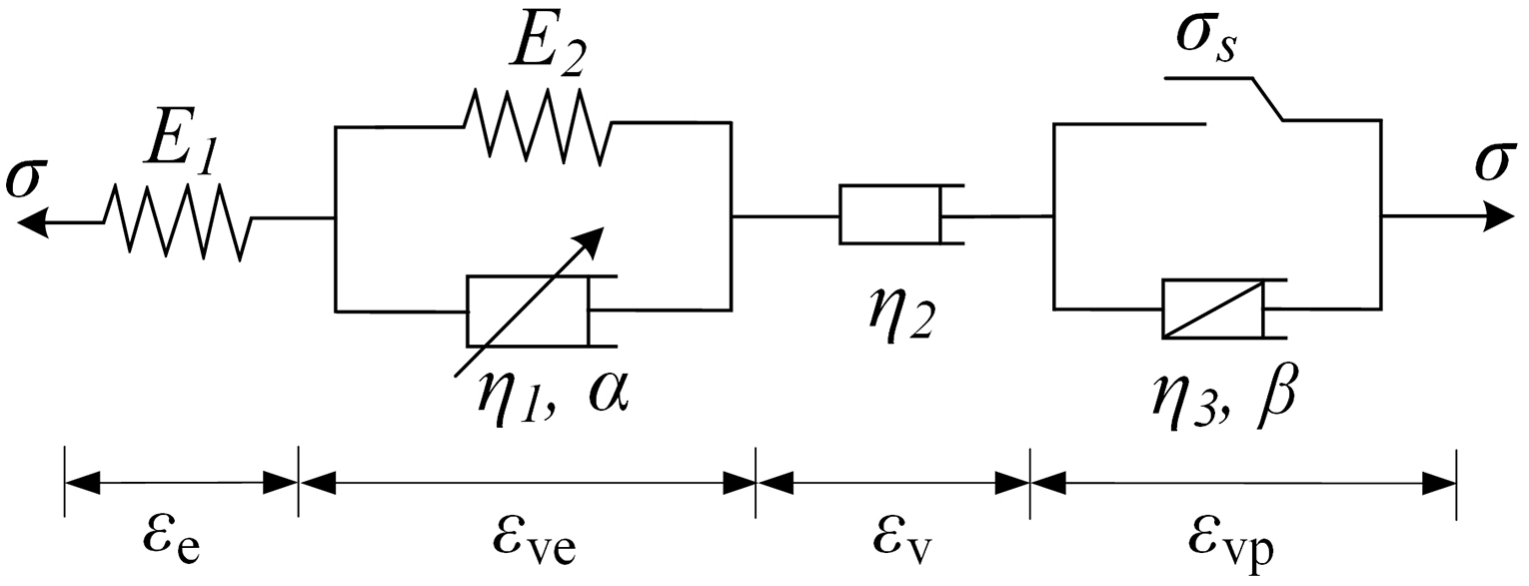
Schematic diagram of nonlinear viscoelastic-plastic creep model of rock.

The instantaneous elastic strain of rock is produced at the moment of loading, which can be described by Hooke’s body. Then the rock enters the attenuation creep stage, and the unsteady creep parameter Kelvin proposed above can be used to describe the characteristics of the rock attenuation creep stage. The constant creep stage can be described by classical viscous elements. When the rock enters the accelerated creep stage, the nonlinear accelerated creep characteristics of the rock can be described by the nonlinear viscoplastic body proposed above. Thus, a nonlinear viscoelastic-plastic creep model of rock can be established, as shown in Fig 4.

When $\dot{\varepsilon }\geq 0,\;\ddot{\varepsilon }<0,\;\sigma <\sigma_{s}$, that is, when the rock undergoes three processes of instantaneous elastic strain, attenuation creep and stable creep, the state equation of the creep model is:


$$\begin{array}{c} \left\{ \begin{array}{c} \sigma =\sigma_{e}=\sigma_{ve}\\ \varepsilon =\varepsilon_{e}+\varepsilon_{ve}\\ \sigma_{e}=E_{\text{1}}\varepsilon_{e}\\ \sigma_{ve}=E_{\text{2}}\varepsilon_{ve}+\eta_{\text{1}}(t/t_{\text{0}}+1{)}^{-\alpha }{\dot{\varepsilon }}_{ve} \end{array}\right. \end{array}$$ (14)

From Equation (14), we can get:


$$\begin{array}{c} \varepsilon =\frac{\sigma }{E_{\text{1}}}+\frac{\sigma }{E_{\text{2}}}\left(1-exp\left(\frac{E_{\text{2}}(1-(t/t_{\text{0}}+1{)}^{\alpha +1})}{\eta_{\text{1}}(\alpha +1)}\right)\right) \end{array}$$ (15)

The Equation (15) is the creep equation of the nonlinear creep model of rock when $\dot{\varepsilon }\geq 0,\;\ddot{\varepsilon }<0,\;\sigma <\sigma_{s}$.

When $\dot{\varepsilon }>0,\;\ddot{\varepsilon }=0,\;\sigma <\sigma_{s}$, that is, when the rock undergoes three processes of instantaneous elastic strain, attenuation creep and constant creep, the state equation of the creep model is:


$$\begin{array}{c} \left\{ \begin{array}{c} \sigma =\sigma_{e}=\sigma_{ve}=\sigma_{v}\\ \varepsilon =\varepsilon_{e}+\varepsilon_{ve}+\varepsilon_{v}\\ \sigma_{e}=E_{\text{1}}\varepsilon_{e}\\ \sigma_{ve}=E_{\text{2}}\varepsilon_{ve}+\eta_{\text{1}}(t/t_{\text{0}}+1{)}^{-\alpha }{\dot{\varepsilon }}_{ve}\\ \sigma_{v}=\eta_{\text{2}}{\dot{\varepsilon }}_{v} \end{array}\right. \end{array}$$ (16)

From Equation (16), we can get:


$$\begin{array}{c} \varepsilon =\frac{\sigma }{E_{\text{1}}}+\frac{\sigma }{E_{\text{2}}}\left(1-exp\left(\frac{E_{\text{2}}(1-(t/t_{\text{0}}+1{)}^{\alpha +1})}{\eta_{\text{1}}(\alpha +1)}\right)\right)+\frac{\sigma }{\eta_{\text{2}}}t \end{array}$$ (17)

The Equation (17) is the creep equation of the nonlinear creep model of rock when $\dot{\varepsilon }>0,\;\ddot{\varepsilon }=0,\;\sigma <\sigma_{s}$.

When $\dot{\varepsilon }>0,\;\ddot{\varepsilon }>0,\;\sigma \geq \sigma_{s}$, that is, when the rock undergoes four processes of instantaneous elastic strain, attenuation creep, constant creep and accelerated creep, the state equation of the creep model is:


$$\begin{array}{c} \left\{ \begin{array}{c} \sigma =\sigma_{e}=\sigma_{ve}=\sigma_{v}=\sigma_{vp}\\ \varepsilon =\varepsilon_{e}+\varepsilon_{ve}+\varepsilon_{v}+\varepsilon_{vp}\\ \sigma_{e}=E_{\text{1}}\varepsilon_{e}\\ \sigma_{ve}=E_{\text{2}}\varepsilon_{ve}+\eta_{\text{1}}(t/t_{\text{0}}+1{)}^{-\alpha }{\dot{\varepsilon }}_{ve}\\ \sigma_{v}=\eta_{\text{2}}{\dot{\varepsilon }}_{v}\\ \sigma_{vp}=\sigma_{s}+\eta_{\text{3}}e^{-\beta (\sigma -\sigma_{s})t}{\dot{\varepsilon }}_{vp} \end{array}\right. \end{array}$$ (18)

From Equation (18), we can get:


$$\begin{array}{c} \varepsilon =\frac{\sigma }{E_{\text{1}}}+\frac{\sigma }{E_{\text{2}}}\left(1-exp\left(\frac{E_{\text{2}}(1-(t/t_{\text{0}}+1{)}^{\alpha +1})}{\eta_{\text{1}}(\alpha +1)}\right)\right)+\frac{\sigma }{\eta_{\text{2}}}t+\frac{\text{1}}{\beta \eta_{\text{3}}}\left(exp\left(\beta \left(\sigma -\sigma_{s}\right)t\right)-1\right) \end{array}$$ (19)

The Equation (19) is the creep equation of the nonlinear creep model of rock when $\dot{\varepsilon }>0,\;\ddot{\varepsilon }>0,\;\sigma \geq \sigma_{s}$.

## 3. Three-dimensional creep equation of creep model

In practical engineering, rock is often in a three-dimensional stress state, and one-dimensional creep equation is difficult to describe the creep behavior of rock under three-dimensional stress state. Therefore, it is necessary to extend the established one-dimensional creep equation to the creep equation under three-dimensional stress state.

Under the three-dimensional stress state, the stress tensor *σ*_*ij*_ of a point in the rock can be decomposed into the spherical stress tensor *σ*_*m*_ and the deviatoric stress tensor *S*_*ij*_, then:


$$\begin{array}{c} \left\{ \begin{array}{c} \sigma_{m}=\frac{\text{1}}{\text{3}}(\sigma_{\text{1}}+\sigma_{\text{2}}+\sigma_{\text{3}})=\frac{\text{1}}{\text{3}}\sigma_{kk}\\ S_{ij}=\sigma_{ij}-\delta_{ij}\sigma_{m}=\sigma_{ij}-\frac{\text{1}}{\text{3}}\delta_{ij}\sigma_{kk} \end{array}\right. \end{array}$$ (20)

In the formula, *δ*_*ij*_ is the Kronecker symbol, and the spherical stress tensor only changes the volume of the object and does not change the shape; the deviatoric stress tensor only changes the shape without changing the volume. Correspondingly, the strain tensor *ε*_*ij*_ of a point in the rock can be decomposed into the spherical strain tensor *ε*_*m*_ and the deviatoric strain tensor *e*_*ij*_, then:


$$\begin{array}{c} \left\{ \begin{array}{c} \varepsilon_{ij}=e_{ij}+\delta_{ij}\varepsilon_{m}\\ \varepsilon_{m}=\frac{\text{1}}{\text{3}}\varepsilon_{kk} \end{array}\right. \end{array}$$ (21)

In the three-dimensional state, it can be obtained by Hooke’s law:


$$\begin{array}{c} \left\{ \begin{array}{c} \sigma_{m}=3K\varepsilon_{m}\\ S_{ij}=2Ge_{ij} \end{array}\right. \end{array}$$ (22)

Where *K* is the bulk modulus and *G* is the shear modulus, the expression is as follows:


$$\begin{array}{c} \left\{ \begin{array}{c} K=\frac{E}{3(1-2\mu )}\\ G=\frac{E}{2(1+\mu )} \end{array}\right. \end{array}$$ (23)

In the formula, *E* is the elastic modulus and *μ* is the Poisson’s ratio.

Therefore, the three-dimensional creep equation of Hooke’s body is:


$$\begin{array}{c} \varepsilon_{ij}^{e}=\frac{\text{1}}{2G_{\text{1}}}S_{ij}+\frac{\text{1}}{3K_{\text{1}}}\delta_{ij}\sigma_{m} \end{array}$$ (24)

The three-dimensional creep equation of unsteady Kelvin body is:


$$\begin{array}{c} \varepsilon_{ij}^{ve}=\frac{S_{ij}}{2G_{\text{2}}}\left(1-exp\left(\frac{G_{\text{2}}\left(1-{\left(t+1\right)}^{\alpha +1}\right)}{\eta_{\text{1}}\left(\alpha +1\right)}\right)\right) \end{array}$$ (25)

The three-dimensional creep equation of Newton body is:


$$\begin{array}{c} \varepsilon_{ij}^{v}=\frac{S_{ij}}{2\eta_{\text{2}}}t \end{array}$$ (26)

The constitutive relation of nonlinear viscoplastic body is:


$$\begin{array}{c} {\dot{\varepsilon }}_{ij}^{vp}=\frac{\text{1}}{\eta_{\text{3}}(1-D)}\langle \Phi \left(\frac{F}{F_{\text{0}}}\right)\rangle \frac{\partial Q}{\partial \sigma_{ij}} \end{array}$$ (27)

In the formula, *F* is the yield function, *F*_*0*_ is the initial state yield function value, generally taken as 1. $\Phi \left(\frac{F}{F_{\text{0}}}\right)$ is a power function, and $\Phi \left(\frac{F}{F_{\text{0}}}\right)={\left(\frac{F}{F_{\text{0}}}\right)}^{n}$, for rock materials, usually take *n* = 1.

⟨⟩ represents the switching function, that is,


$$\begin{array}{c} \langle \Phi \left(\frac{F}{F_{\text{0}}}\right)\rangle =\left\{ \begin{array}{c} 0\text{\textasciitilde{} }(F<0)\\ \Phi \left(\frac{F}{F_{\text{0}}}\right)\text{ (}F\geq 0) \end{array}\right. \end{array}$$ (28)

Q is the plastic potential function. For the convenience of calculation, the associated flow rule is adopted [45], that is, F=Q.

The rock yield criteria mainly include Mohr-Coulomb yield criterion, Von-Mises yield criterion and Drucker-Prager yield criterion. Among them, Mohr-Coulomb yield criterion can not consider the influence of intermediate principal stress on rock yield, Von-Mises yield criterion ignores the influence of spherical stress on rock creep characteristics, and Drucker-Prager yield criterion can make up for the shortcomings of the above two criteria. Therefore, Drucker-Prager yield criterion is adopted in this paper. Its expression is,


$$\begin{array}{c} F=\sqrt{J_{\text{2}}}-\mu I_{\text{1}}-k \end{array}$$ (29)

In the formula, $J_{\text{2}}$ is the second invariant of stress deviator, $I_{\text{1}}$ is the first invariant of stress tensor, *μ* and *k* are material parameters.


$$\begin{array}{c} \left\{ \begin{array}{c} \mu =\frac{sin\phi }{\sqrt{3(3+{sin}^{\text{2}}\phi )}}\\ k=\frac{\sqrt{\text{3}}c\;cos\phi }{\sqrt{3+{sin}^{\text{2}}\phi }} \end{array}\right. \end{array}$$ (30)

In the formula, *φ* is the internal friction angle of rock material, and *c* is the cohesion of rock material.

By combining Equations (20) to (30) and applying the principle of superposition, the creep equation of the rock under three-dimensional stress state is obtained as:


$$\begin{array}{c} \varepsilon =\left\{ \begin{array}{c} \frac{\sigma_{\text{1}}+2\sigma_{\text{3}}}{9K_{\text{1}}}+\frac{\sigma_{\text{1}}-\sigma_{\text{3}}}{3G_{\text{1}}}+\frac{\sigma_{\text{1}}-\sigma_{\text{3}}}{3G_{\text{2}}}\left(1-exp\left(\frac{G_{\text{2}}\left(t_{\text{0}}^{\alpha +1}-{\left(t+t_{\text{0}}\right)}^{\alpha +1}\right)}{\eta_{\text{1}}t_{\text{0}}^{\alpha }\left(\alpha +1\right)}\right)\right)\;\;(\dot{\varepsilon }\geq 0,\;\ddot{\varepsilon }<0,\;\sigma <\sigma_{s})\\ \frac{\sigma_{\text{1}}+2\sigma_{\text{3}}}{9K_{\text{1}}}+\frac{\sigma_{\text{1}}-\sigma_{\text{3}}}{3G_{\text{1}}}+\frac{\sigma_{\text{1}}-\sigma_{\text{3}}}{3G_{\text{2}}}\left(1-exp\left(\frac{G_{\text{2}}\left(t_{\text{0}}^{\alpha +1}-{\left(t+t_{\text{0}}\right)}^{\alpha +1}\right)}{\eta_{\text{1}}t_{\text{0}}^{\alpha }\left(\alpha +1\right)}\right)\right)\\ +\frac{\sigma_{\text{1}}-\sigma_{\text{3}}}{3\eta_{\text{2}}}t\text{ (}\dot{\varepsilon }>0,\;\ddot{\varepsilon }=0,\;\sigma <\sigma_{s})\\ \frac{\sigma_{\text{1}}+2\sigma_{\text{3}}}{9K_{\text{1}}}+\frac{\sigma_{\text{1}}-\sigma_{\text{3}}}{3G_{\text{1}}}+\frac{\sigma_{\text{1}}-\sigma_{\text{3}}}{3G_{\text{2}}}\left(1-exp\left(\frac{G_{\text{2}}\left(t_{\text{0}}^{\alpha +1}-{\left(t+t_{\text{0}}\right)}^{\alpha +1}\right)}{\eta_{\text{1}}t_{\text{0}}^{\alpha }\left(\alpha +1\right)}\right)\right)\\ +\frac{\sigma_{\text{1}}-\sigma_{\text{3}}}{3\eta_{\text{2}}}t+\frac{\text{1}}{3\beta \eta_{\text{3}}}\left(exp\left(\beta \left(\sigma_{\text{1}}-\sigma_{\text{3}}-\sigma_{s}\right)t\right)-1\right)\;\;\text{ (}\dot{\varepsilon }>0,\;\ddot{\varepsilon }>0,\;\sigma \geq \sigma_{s}) \end{array}\right. \end{array}$$ (31)

## 4. Verification of creep model

**Fig 5:**
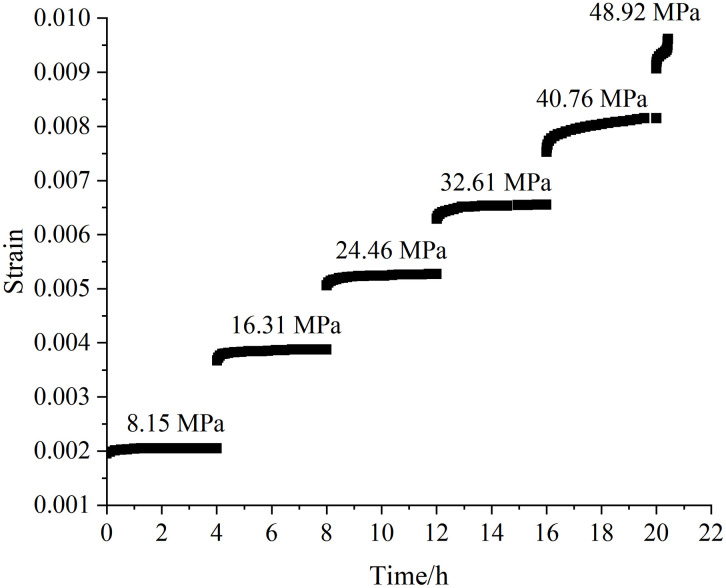
Creep test results of sandstone under confining pressure of 0 MPa.

**Fig 6:**
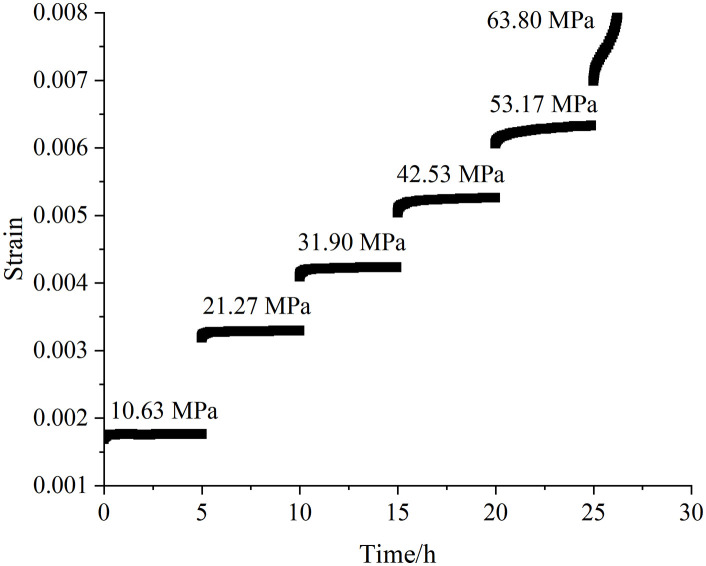
Creep test results of sandstone under confining pressure of 5 MPa.

In this paper, the creep test data of sandstone by Liu et al. [46] are used to verify the rationality and applicability of the model established in this paper. In this paper, the test data of confining pressure of 0 MPa and 5 MPa are selected. The test conditions are shown in Table 1, and the test results are shown in Figs 5 and 6.

**Table 1:** Test conditions.

Confining pressure/ MPa	All levels of load/ MPa					
0	8.15	16.31	24.46	32.61	40.76	48.92
5	10.63	21.27	31.90	42.53	53.17	63.80

**Fig 7:**
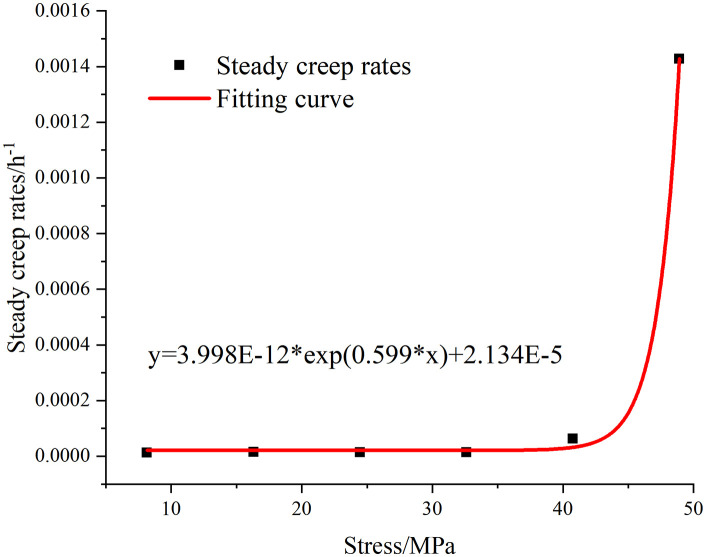
The relationship between steady creep rate and shear stress at Confining pressure of 0 MPa.

**Fig 8:**
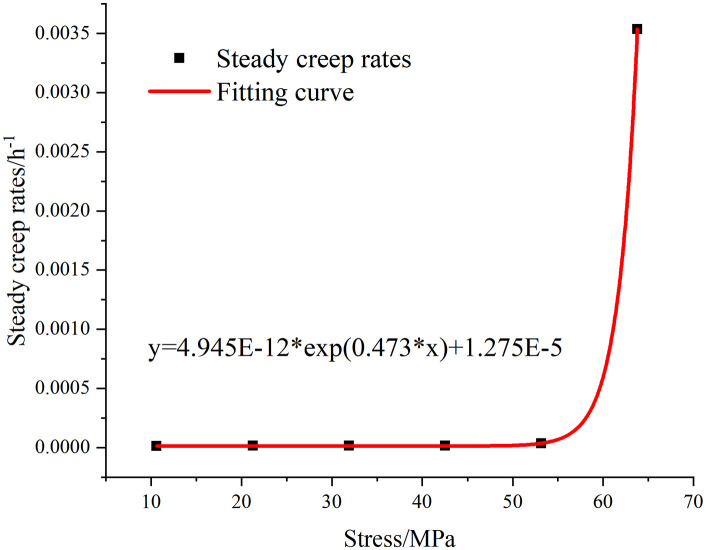
The relationship between steady creep rate and shear stress at Confining pressure of 5 MPa.

The long-term strength of sandstone needs to be known when the model is verified. According to the above test data, the long-term strength can be obtained by the steady-state creep rate method. The specific method is as follows: through the results of sandstone creep test, the creep rate of sandstone is obtained, and the relationship curve between steady-state creep rate and stress is drawn, as shown in Figs 7 and 8. The stress value corresponding to the sudden change point of steady-state creep rate is taken as the long-term strength of sandstone.

As shown in Figs 7 and 8, the steady creep rate is in an exponential function relationship with the stress. It was fitted using the least squares method, and the results are presented in Equations (32) and (33).


$$\begin{array}{c} y=3.998\text{E}-12*exp(0.599*x)+2.134\text{E}-5 \end{array}$$ (32)

$$\begin{array}{c} y=4.945\text{E}-12*exp(0.473*x)+1.275\text{E}-5 \end{array}$$ (33)

Taking the derivatives of Equations (32) and (33), the expression for the slope *K* can be obtained as:


$$\begin{array}{c} K=y^{\text{'}}=3.998\text{E}-12*0.599*exp(0.599*x) \end{array}$$ (34)

$$\begin{array}{c} K=y^{\text{'}}=4.945\text{E}-12*0.473*exp(0.473*x) \end{array}$$ (35)

When the slope is K≤1, the steady creep rate increases slowly. When the slope is K>1, the steady creep rate increases significantly faster. Therefore, the creep rate before and after the corresponding point at K=1 changes greatly. This point is a sudden change point, and the corresponding stress value is the long-term strength of the rock. It can be determined that the long-term strength of sandstone under confining pressure of 0 MPa and 5 MPa is 44.67 MPa and 56.62 MPa, respectively.

In order to verify the reliability of the method, we also used the stress-strain isochronous curve method to obtain the long-term strength of sandstone at confining pressure of 0 MPa and 5 MPa, which were 40.76 MPa and 53.17 MPa, respectively, which was not much different from the results obtained by the steady-state rate method, which confirmed the reliability of our method.

**Fig 9:**
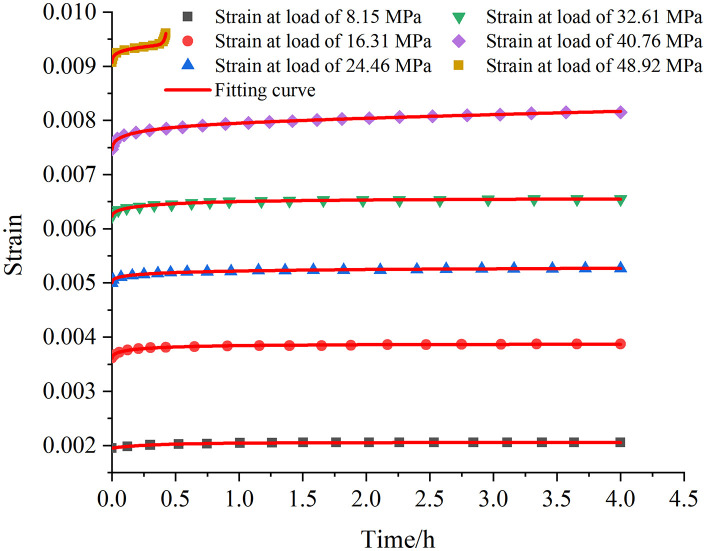
Fitting curve at confining pressure of 0 MPa.

When the confining pressure is 0 MPa, the sandstone is in the uniaxial compression state. Under the first four levels of load, the steady-state creep rate is close to 0, and the Equation (15) can be used to fit the test data. Under the fifth level of load, the steady-state creep rate is not 0, and the Equation (17) can be used to fit the test data. Under the sixth level of load, the sandstone will enter the accelerated creep stage, and the Equation (19) can be used to fit the test data. The fitting curve is shown in Fig 9, and the creep model parameters obtained by fitting are shown in Table 2.

**Table 2:** Creep model parameters at confining pressure of 0 MPa.

Stress/MPa	*E*_1_/GPa	*E*_2_/GPa	*α*	*η*_1_/GPa·h	*η*_2_/GPa·h	*β*	*η*_3_/GPa·h	R^2^	RMSE	MAE
8.15	4.108	81.665	0.844	40.104	—	—	—	0.995	0.0023	0.0018
16.31	4.740	64.519	0.465	62.081	—	—	—	0.988	0.0041	0.0033
24.46	4.652	80.996	0.387	172.934	—	—	—	0.996	0.0019	0.0015
32.61	5.345	98.381	0.520	114.959	—	—	—	0.997	0.0015	0.0012
40.76	5.647	82.739	0.489	81.346	758.496	—	—	0.996	0.0018	0.0014
48.92	5.297	186.118	0.452	94.767	270.250	12.689	221.879	0.981	0.0052	0.0041

**Fig 10:**
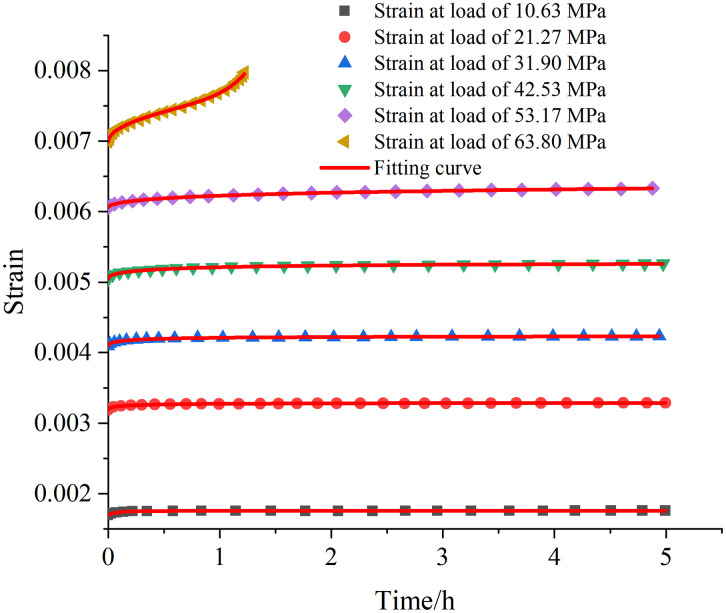
Fitting curve at confining pressure of 5 MPa.

When the confining pressure is 5 MPa, the sandstone is in the triaxial compression state of equal confining pressure. Under the first four loads, the steady-state creep rate is close to 0, and the first equation of Equation (31) can be used to fit the test data. Under the fifth load, the steady-state creep rate is not 0, and the second equation of Equation (31) can be used to fit the test data. Under the sixth load, the sandstone will enter the accelerated creep stage, and the third equation of Equation (31) can be used to fit the test data. The fitting curve is shown in Fig 10, and the creep model parameters obtained by fitting are shown in Table 3.

**Table 3:** Creep model parameters at confining pressure of 5 MPa.

Stress/MPa	*K*_1_/GPa	*G*_1_/GPa	*G*_2_/GPa	*α*	*η*_1_/GPa·h	*η*_2_/GPa·h	*β*	*η*_3_/GPa·h	R^2^	RMSE	MAE
10.63	10.277	1.282	30.175	0.753	5.736	—	—	—	0.989	0.0031	0.0025
21.27	11.863	1.969	54.163	0.441	62.048	—	—	—	0.991	0.0028	0.0022
31.90	12.770	2.291	60.535	0.443	75.599	—	—	—	0.992	0.0025	0.0020
42.53	14.210	2.604	55.328	0.474	90.936	—	—	—	0.997	0.0015	0.0011
53.17	14.862	2.949	71.264	0.564	108.579	1338.704	—	—	0.994	0.0021	0.0017
63.80	15.280	3.089	19.467	0.505	55.571	458.839	1.120	966.196	0.987	0.0048	0.0039

The parameter identification of the model is performed using the nonlinear least squares fitting algorithm in Origin. The initial parameter settings are based on typical rock mechanics values and boundaries are set to ensure physical rationality. The convergence criterion is set to a tolerance of 1e-6.

It can be seen from Figs 9 and 10 that the fitting curve of the model is in good agreement with the test curve, and it can also be seen from Table 2 and 3 that the value of R^2^ is basically above 0.98, and the RMSE/MAE value is very small. It shows that the model established in this paper can well describe the creep characteristics of rock under one-dimensional stress state or three-dimensional stress state, thus verifying the rationality and applicability of the creep model.

It should be noted that the three-dimensional creep equation has been verified by conventional triaxial compression data. Although this is a common verification method, we admit that it does not fully examine the performance of the model under general three-dimensional stress states, such as real triaxial conditions. Comprehensive verification under such conditions is the direction of future research. However, the Drucker-Prager criterion used in the model itself takes into account the influence of intermediate principal stress and spherical stress, which makes the framework more comprehensive than those models using simpler criteria. The model ‘s ability to handle more complex loading paths has thus become an important theoretical advantage.

**Fig 11:**
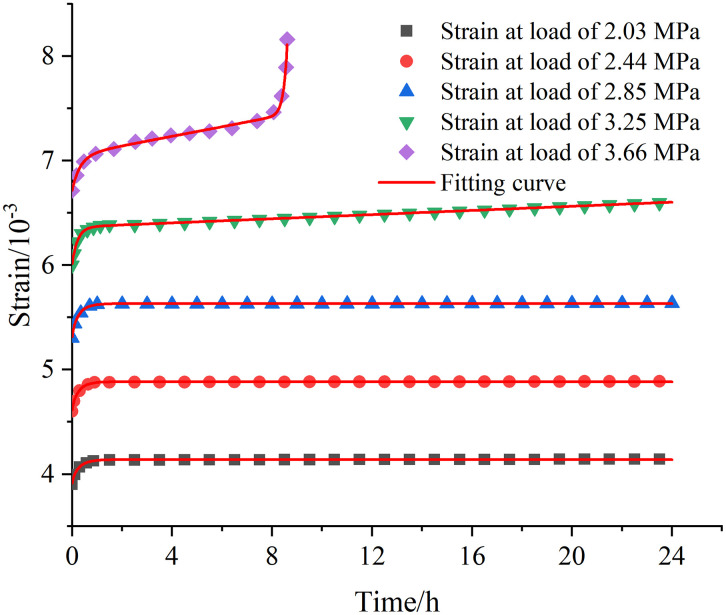
Creep test fitting curve of carbonaceous shale.

In order to verify whether the model is suitable for other types of rocks, the above method is used to fit and analyze the creep test data of carbonaceous shale in reference [47]. The results are shown in Fig 11 and Table 4.

**Table 4:** Creep model parameters of carbonaceous shale.

Stress/MPa	*E*_1_/GPa	*E*_2_/GPa	*α*	*η*_1_/GPa·h	*η*_2_/GPa·h	*β*	*η*_3_/GPa·h	R^2^	RMSE	MAE
2.03	0.521	8.892	0.864	3.109	—	—	—	0.995	0.0015	0.0012
2.44	0.526	9.020	0.903	2.803	—	—	—	0.997	0.0012	0.0010
2.85	0.538	8.991	0.889	2.920	—	—	—	0.993	0.0018	0.0014
3.25	0.539	9.180	1.001	2.022	—	—	—	0.996	0.0014	0.0011
3.66	0.548	10.557	0.943	3.784	80.039	20.404	3549.626	0.991	0.0032	0.0026

From Fig 11, it can be seen that the model fitting curve and the experimental curve are in good agreement, and the R^2^ values in Table 4 are basically close to 1, and the RMSE/MAE values are very small, indicating that the model established in this paper is also suitable for describing the creep process characteristics of carbonaceous shale, further indicating that the model established in this paper has a wide range of applicability.

It is worth pointing out that the model parameters in this paper are equivalent fitting parameters under specific stress levels, and their changes with stress levels reflect the stress dependence of rock creep behavior, rather than model defects. Establishing a quantitative relationship between parameters and stress levels will be the focus of subsequent research.

**Fig 12:**
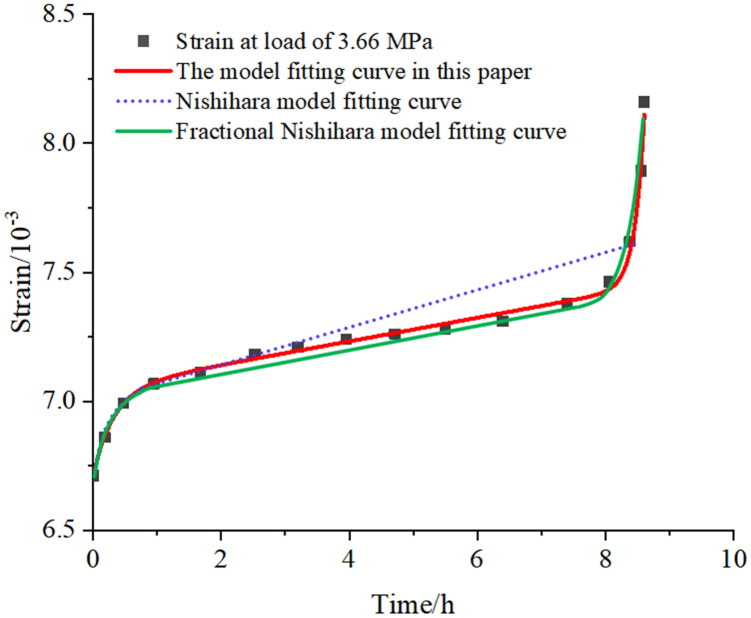
Fitting curves of different models.

In order to further demonstrate the predictive ability of the model, we conducted a comparative analysis with the classical Nishihara model and the fractional Nishihara model. The creep test data of carbonaceous shale at a stress of 3.66 MPa are used for comparative analysis. The results are shown in Fig 12 and Table 5.

**Table 5:** Comparison of fitting effects of different models.

Model	R^2^	RMSE	MAE
Model in this paper	0.991	0.0032	0.0026
Nishihara model	0.791	0.0970	0.0754
Fractional Nishihara model	0.983	0.0195	0.0152

It can be seen from Fig 12 and Table 5 that the model in this paper is significantly better than the classical Nishihara model, which fails to describe the accelerated creep stage of rock. In addition, the model in this paper also shows obvious advantages over the fractional Nishihara model, achieving higher R^2^ value and lower RMSE/MAE value. This shows that the model in this paper can better describe the creep process of rock.

## 5. Conclusions

A reasonable rock creep model is of great significance for analyzing the creep characteristics of rock and predicting the long-term stability of engineering rock mass. In this paper, through in-depth theoretical research, a rock creep model based on creep parameter degradation is innovatively constructed, and the model is verified by experimental data. The main conclusions are as follows:

(1) Based on the analysis of the nonlinear creep characteristics of rock, the unsteady Kelvin body reflecting the attenuation creep characteristics and the nonlinear viscoplastic body reflecting the accelerated creep characteristics are innovatively proposed in this paper. The viscoelastoplastic creep model of rock based on the deterioration of creep parameters is established, and the creep equations of the creep model under one-dimensional stress state and three-dimensional stress state are derived.
(2) Using the model proposed in this paper, the creep test data of sandstone under different confining pressures and the creep test data of carbonaceous shale are fitted and analyzed respectively. The results show that the experimental curve and the model curve are in good agreement, with the coefficient of determination R^2^ generally above 0.98 and low RMSE/MAE values, indicating that the nonlinear viscoelastic-plastic creep model of rock established in this paper can well describe the creep characteristics of rock. At the same time, the fitting effects of the model, the classical Nishihara model and the fractional Nishihara model are compared to further illustrate the correctness and advancement of the established model.
(3) The nonlinear viscoelastic-plastic creep model of rock can provide a theoretical basis for predicting the trend of creep deformation of rock mass engineering such as water conservancy and hydropower engineering, tunnel engineering, deep underground roadway and urban underground engineering over time, and provide potential reference value for long-term stability analysis. However, there are still some limitations in the current model, such as the need to verify under complex load conditions and a large number of creep data, which is also our next research direction.

## References

[1] Aghajanzadeh M, Li D, Heidarpour A, Bahaaddini M, Masoumi H. Shape-dependent behavior of intact rock under creep loading. Rock Mech Rock Eng. 2025;58(4):4225–45. doi: 10.1007/s00603-024-04358-8

[2] Cyran K, Kowalski M. Effect of pillar width on the stability of the salt cavern field for energy storage. Studia Geotechnica et Mechanica. 2024;46(3):147–63. doi: 10.2478/sgem-2024-0009

[3] Li Q, Li QC, Wang FL, Wu JJ, Wang YL, Jin JF. Effects of geological and fluid characteristics on the injection filtration of hydraulic fracturing fluid in the wellbores of shale reservoirs: numerical analysis and mechanism determination. Processes. 2025;13(6):1747. doi: 10.3390/pr13061747

[4] Marmoni GM, Martino S, Censi M, Menichetti M, Piacentini D, Scarascia MG. Transition from rock mass creep to progressive failure for rockslide initiation at Mt. Conero (Italy). Geomorphology. 2023;437:108750. doi: 10.1016/j.geomorph.2023.108750

[5] Delchiaro M, Seta MD, Martino S, Moumeni M, Nozaem R, Marmoni GM. The role of long-term preparatory factors in mass rock creep deforming slopes: insights from the Zagros Mts. belt (Iran). Landslides. 2024;21(8):1735–55. doi: 10.1007/s10346-024-02252-6

[6] Kovrizhnykh AM, Baryshnikov VD, Khmelinin AP. Determining time-to-failure in rocks using long-term strength criterion. J Min Sci. 2023;59(6):911–8. doi: 10.1134/s1062739123060042

[7] Tarifard A, Török Á, Görög P. Review of the creep constitutive models for rocks and the application of creep analysis in geomechanics. Rock Mech Rock Eng. 2024;57(10):7727–57. doi: 10.1007/s00603-024-03939-x

[8] Cirone A, Vargas E Jr. A thermodynamic modeling of creep in rock salt. Int J Rock Mech Mining Sci. 2023;162:105298. doi: 10.1016/j.ijrmms.2022.105298

[9] Li Q, You D, Li Q, Wang F, Wang Y, Yang Y. Analysis of sedimentation behavior and influencing factors of solid particles in CO2 fracturing fluid. Processes. 2025;13(12):4049. doi: 10.3390/pr13124049

[10] AlDhuhoori M, Belhaj H, AlHameli F, Aljaberi F. Assessment of mineral compositions on geo-mechanical time dependent plastic creep deformation. Int J Hydrogen Energy. 2024;83:472–90. doi: 10.1016/j.ijhydene.2024.08.032

[11] Li Q, Li Q, Wu J, He K, Xia Y, Liu J, et al. Wellhead stability during development process of hydrate reservoir in the Northern South China sea: sensitivity analysis. Processes. 2025;13(6):1630. doi: 10.3390/pr13061630

[12] Leone T, Nordas AN, Anagnostou G. An estimation equation for the TBM thrust force in creeping rock. Comput Geotech. 2024;165:105802. doi: 10.1016/j.compgeo.2023.105802

[13] Taheri SR, Pak A, Shad S, Mehrgini B, Razifar M. Investigation of rock salt layer creep and its effects on casing collapse. Int J Min Sci Technol. 2020;30(3):357–65. doi: 10.1016/j.ijmst.2020.02.001

[14] Adamuszek M, Tămaş DM, Barabasch J, Urai JL. Rheological stratification in impure rock salt during long-term creep: morphology, microstructure, and numerical models of multilayer folds in the Ocnele Mari salt mine, Romania. Solid Earth. 2021;12(9):2041–65. doi: 10.5194/se-12-2041-2021

[15] Truster TJ, Moslehy A, Alshibli KA. Effects of crystal orientation, temperature, deviatoric stress, and confining stress on creep of rock salt. Int J Rock Mech Min Sci. 2024;183:105913. doi: 10.1016/j.ijrmms.2024.105913

[16] Wei E, Hu B, Li J, Cui K, Zhang Z, Cui A, et al. Nonlinear viscoelastic‐plastic creep model of rock based on fractional calculus. Adv Civ Eng. 2022;2022(1). doi: 10.1155/2022/3063972

[17] Meng L, Zhang S, Li T, Liu T, Li H. Study on the creep constitutive model of layered rockconsidering anisotropic and damage factors after hightemperature exposure. Int J Damage Mech. 2024;34(3):520–41. doi: 10.1177/10567895241292748

[18] Carlson G, Shirzaei M, Ojha C, Werth S. Subsidence-derived volumetric strain models for mapping extensional fissures and constraining rock mechanical properties in the San Joaquin Valley, California. J Geophys Res Solid Earth. 2020;125(9):e2020JB019980. doi: 10.1029/2020JB019980 33042724 PMC7539920

[19] Zhao TB, Zhang YB, Zhang QQ, Tan YL. Analysis on the creep response of bolted rock using bolted burgers model. Geomech Eng. 2018;14(2):141–9. doi: 10.12989/gae.2018.14.2.141

[20] Zhang L, Wang X. Study on nonlinear damage creep model for rocks under cyclic loading and unloading. Adv Mater Sci Eng. 2021;2021(1). doi: 10.1155/2021/5512972

[21] Kamdem TC, Richard KG, Béda T. New description of the mechanical creep response of rocks by fractional derivative theory. Appl Math Modell. 2023;116:624–35. doi: 10.1016/j.apm.2022.11.036

[22] Liu Z, Oh H, Wu M, Yu K, Zhou H. Creep behavior of mélange rocks and the hardening-damage nonlinear elastic-viscous model. Pure Appl Geophys. 2025;182(2):587–608. doi: 10.1007/s00024-024-03635-5

[23] Martín LB, Rouabhi A, Hassen FH, Jaworowicz J, Azabou M, Hévin G. Creep of rock salt under a large range of deviatoric stresses. Rock Mech Rock Eng. 2024;57(8):6105–18. doi: 10.1007/s00603-024-03841-6

[24] Wang R, Liu L, Zhu M, Qiu H, Tu B, Qu H, et al. Assessing ground stability of a vertical backfilled stope considering creep behaviors of surrounding rocks. J Rock Mech Geotech Eng. 2025;17(1):187–99. doi: 10.1016/j.jrmge.2023.12.010

[25] Cleja-Ţigoiu S, Ţigoiu V. Rheological model for rock-type materials under large deformations. Mech Res Commun. 2021;114:103559. doi: 10.1016/j.mechrescom.2020.103559

[26] Liu D, Shan RL, Wang H, Li Z, Tong X, Zhao Y, et al. Study on triaxial creep mechanical properties and creep model of sandy mudstone with initial radial depth damage. Construct Build Mater. 2025;483:141772. doi: 10.1016/j.conbuildmat.2025.141772

[27] Yuan Y, Xu T, Meredith PG, Mitchell TM, Heap MJ, Heng Z. A time-dependent microplane model for deformation and fracturing of brittle rock. Comput Geotechn. 2025;184:107278. doi: 10.1016/j.compgeo.2025.107278

[28] Huang JH, Hu SC, Li XL, Guo SH, Zhang CX, Gao ZH. Creep model of weakly cemented soft rock considering damage and secondary development in FLAC3D. Appl Sci. 2025;15(9):4838. doi: 10.3390/app15094838

[29] Son MS, Lim ST, Cho KH. Creep prediction of rock embankment in railway roadbed using Kelvin model. J Korean Soc Railway. 2018;21(6):593–603. doi: 10.7782/JKSR.2018.21.6.593

[30] Wang M, Cai M. Simulation of time-dependent response of jointed rock masses using the 3D DEM-DFN modeling approach. Int J Rock Mech Mining Sci. 2025;188:106062. doi: 10.1016/j.ijrmms.2025.106062

[31] Aghajanzadeh M, Masoumi H, Heidarpour A, Alejano LR. Estimating time-to-failure and long-term strength of rocks based on creep strain rate model. Rock Mech Rock Eng. 2024;58(4):4207–23. doi: 10.1007/s00603-024-04088-x

[32] Parui C, Bhattacharyya K. Estimating accurate and precise strain in low-magnitude penetratively deformed rocks: Integrating forward strain modeling and natural data. J Struct Geol. 2024;181:105097. doi: 10.1016/j.jsg.2024.105097

[33] Frenelus W, Peng H, Zhang J. Creep behavior of rocks and its application to the long-term stability of deep rock tunnels. Appl Sci. 2022;12(17):8451. doi: 10.3390/app12178451

[34] Yang S-Q, Xu P, Ranjith PG. Damage model of coal under creep and triaxial compression. Int J Rock Mech Mining Sci. 2015;80:337–45. doi: 10.1016/j.ijrmms.2015.10.006

[35] Farhat F, Shen WQ, Shao JF. A micro-mechanics based viscoplastic model for clayey rocks. Comput Geotech. 2017;89:92–102. doi: 10.1016/j.compgeo.2017.04.014

[36] Hu B, Wei EJ, Li J, Zhu X, Tian KY, Cui K. Nonlinear creep model based on shear creep test of granite. Geomech Eng. 2021;27(5):527–35. doi: 10.12989/gae.2021.27.5.527

[37] Firme PALP, Roehl D, Romanel C. An assessment of the creep behaviour of Brazilian salt rocks using the multi-mechanism deformation model. Acta Geotech. 2016;11(6):1445–63. doi: 10.1007/s11440-016-0451-y

[38] Zhu YG, Wang XY, Liu B, Liu XW, Xue HY, Geng Z. Transversely isotropic creep damage constitutive model for layered rocks. Rock Soil Mech. 2025;46(4):1095–108. doi: 10.16285/j.rsm.2024.1139

[39] Wang ZQ, Hu B, Li J, Chen KK, Wei EJ, Ma LY. Study on creep damage model of soft rock under the influence of dynamic disturbance effect. Chin J Rock Mech Eng. 2024;43(S1):3229–42. doi: 10.13722/j.cnki.jrme.2023.0096

[40] Nazary Moghadam S, Nazokkar K, Chalaturnyk RJ, Mirzabozorg H. Parametric assessment of salt cavern performance using a creep model describing dilatancy and failure. Int J Rock Mech Min Sci. 2015;79:250–67. doi: 10.1016/j.ijrmms.2015.06.012

[41] Chen L, Han J, Zhang S, Jin J, Jia B, Liu J, et al. Creep failure characteristics and damage creep model of red layer soft rock based on Perzyna viscoplastic theory. Sci Rep. 2025;15(1):11084. doi: 10.1038/s41598-025-96352-5 40169871 PMC11962095

[42] Nedjar B, Le Roy R. An approach to the modeling of viscoelastic damage. Application to the long‐term creep of gypsum rock materials. Int J Num Anal Meth Geomechanics. 2012;37(9):1066–78. doi: 10.1002/nag.1138

[43] Nazary Moghadam S, Mirzabozorg H, Noorzad A. Modeling time-dependent behavior of gas caverns in rock salt considering creep, dilatancy and failure. Tunnelling Underground Space Technol. 2013;33:171–85. doi: 10.1016/j.tust.2012.10.001

[44] Wang C, Liu J, Cai Y, Chen L, Wu Z, Liu J. Effects of damage and fractional derivative operator on creep model of fractured rock. Rock Mech Rock Eng. 2024;57(11):9323–41. doi: 10.1007/s00603-024-04061-8

[45] Nazary MS, Mirzabozorg H, Noorzad A. Modeling time-dependent behavior of gas caverns in rock salt considering creep, dilatancy and failure. Tunnell Underground Space Technol. 2013;33:171–85. doi: 10.1016/j.tust.2012.10.001

[46] Liu DY, Xie LJ, Tuo XF, Long LJ. Creep properties of sandstone under different confining pressures and research on a nonlinear viscoelasto-plastic creep model. Chin J Geotech Eng. 2017;36(S2):3705–12. doi: 10.13722/j.cnki.jrme.2017.0023

[47] Wei E, Hu B, Li J, Zhang Z, Ma L, Wang Z. Study on creep mechanical properties of carbonaceous shale under dry-wet cycle. Phys Scr. 2023;98(9):095022. doi: 10.1088/1402-4896/ace742

